# Mastoiditis secondary to mycobacterium abscessus imaged with gallium-67 scintigraphy

**DOI:** 10.2349/biij.4.2.e23

**Published:** 2008-04-01

**Authors:** A Vijayananthan, AV Arumugam, G Kumar, D Harichandra

**Affiliations:** Department of Biomedical Imaging, University of Malaya, Kuala Lumpur, Malaysia

**Keywords:** Mastoiditis, Mycobacterium abscessus, gallium-67 scintigraphy

## Abstract

Atypical mycobacterium is rarely seen as a cause of chronic mastoiditis but has been increasingly recognized over the past few years. *Mycobacterium abscessus* is the most pathogenic and chemotherapy-resistant, rapid-growing mycobacterium of all the four groups. This paper presents a case of a 57-year-old woman who had chronic mastoiditis with recurrent exacerbations. The initial computed tomography (CT) findings showed the presence of an inflammatory process and she was treated with the appropriate antibiotics. The patient subsequently underwent a tissue biopsy when she presented with another exacerbation. At this time, the CT scan did not identify the ongoing exacerbation, but the Gallium-67 scintigraphy did.

## INTRODUCTION

Atypical mycobacterium is a rare entity implicated in *de novo* mastoiditis. There are very few reported cases especially in the South-East Asian region. Various strains of atypical mycobacterium have been known to cause disease in human beings, since their isolation in the 1920s. Of the four groups of atypical mycobacterium, the group IV rapid growers (*Mycobacterium abscessus* and *Mycobacterium fortuitum*) are the ones which are recognized as human pathogens [1].

This paper presents a case of a 57-year-old woman who had chronic recurring mastoiditis and discusses the computed tomography (CT) findings and the use of the Gallium-67 scintigraphy in this condition.

## CASE REPORT

A 57-year-old woman, presented to the ENT clinic with gradual loss of hearing and bilateral ear discharge. She also noticed that pieces of bone were extruding from her left ear. She had been suffering from chronic bilateral ear discharge since her early teenage years. The physical examination done by the otorhinolaryngologist had shown some ear discharge, but there was no evidence of abscess formation, nor was there any facial nerve palsy noted. A presumptive diagnosis of bilateral chronic suppurative otitis media was made. Her blood investigations were essentially normal apart from the normocytic normochromic anemia. Ear swabs from the external auditory canal were sent for microbiological analysis (culture and sensitivity) for pathogens, including *Mycobacterium tuberculosis*.

**Figure 1:**
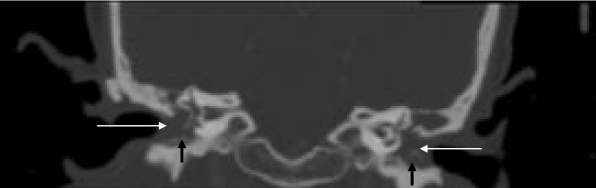
CT scan of temporal bone - Coronal reconstruction image showing soft tissue filling up the external auditory canal and the middle ear (long white arrows). There are also bony fragments seen (short black arrows).

**Figure 2:**
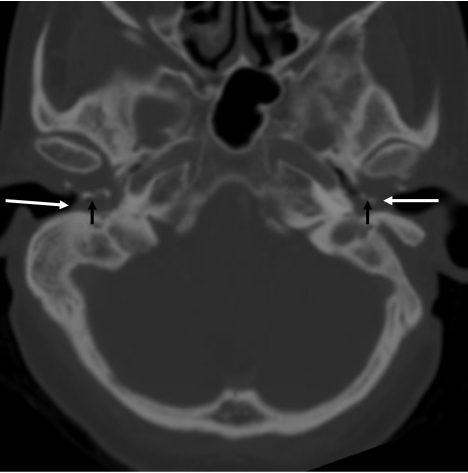
CT of temporal bone - axial image, in bone window showing soft tissue filling up the external auditory canal and the middle ear (long white arrows). The bony fragments are clearly seen in this window. (short black arrows).

A CT scan of the temporal bones revealed sclerosis of both mastoid air cells with presence of soft tissue, bony erosion and bony fragments within the external auditory canal (Figures 1 and 2). She was started on treatment with oral co-trimoxazole because the initial clinical diagnosis prior to the imaging studies was chronic suppurative otitis media. Her ear discharge resolved and her hearing improved. The cultures were negative for pathogens, including tuberculosis. After completing two months of oral antibiotics, she began to notice discharge from her right ear. Due to the recurrent nature of the infection, a tissue biopsy of the ear was performed and sent for culture and sensitivity screening.

The tissue biopsy of the affected ear isolated a rapidly growing atypical mycobacterium – *Mycobacterium abscessus* which was resistant to co-trimoxazole (Bactrim) but sensitive to imipenem (intravenous beta-lactam antibiotics), clarithromycin and azithromycin. She was commenced on imipenem and clarithromycin. Intravenous amikacin was administered initially for synergistic reasons but the patient was unable to tolerate amikacin and it was withheld. This dual antibiotic regime was continued for a total of 40 days without any complications.

**Figure 3:**
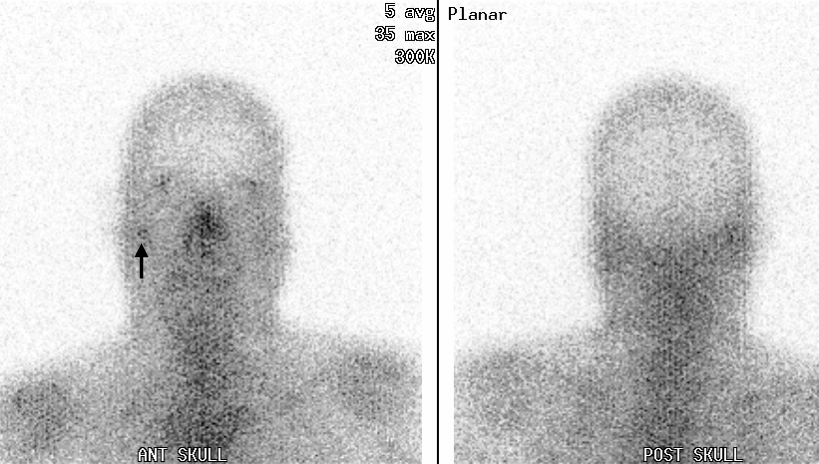
^67^Ga scintigraphy showing uptake in the right mastoid region. (black arrow).

**Figure 4:**
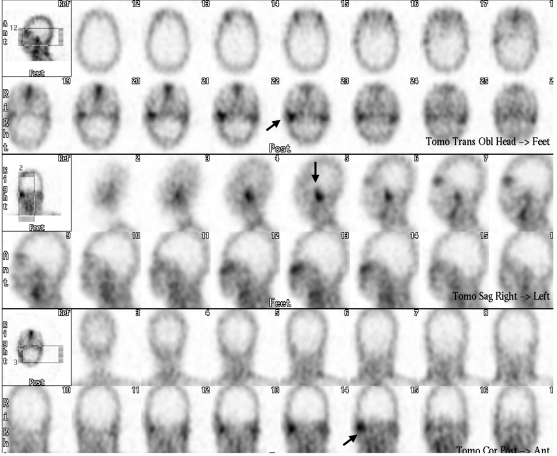
SPECT ^67^Ga scintigraphy images showing significantly increased uptake in the right petromastoid region. (black arrow).

A repeat CT scan of the temporal bone showed consolidation of the previously inflamed bony lesions but was unable to give any information on the activity of infection. A Gallium-67 (^67^Ga) scan was performed and showed raised ^67^Ga uptake in the right petromastoid region (Figures 3 and 4). The left petromastoid region showed near normal, but slightly increased radiotracer uptake. This indicated the presence of active infection and the need to continue treatment.

Upon completing the initial regime of antibiotics, she was started on oral doxycycline 100mg bd, clarithromycin 500mg bd and moxifloxacin 400mg bd for life. She has been on regular follow up since then and has been well.

## DISCUSSION

Mycobacteria are slow-growing aerobic, immobile bacilli that have been identified by the property of acid-fastness. Atypical or non-tuberculous Mycobacteria have been distinguished from the *Mycobacterium tuberculosis* complex and classified into four groups by Runyon based on the speed of growth, morphology and biochemical reactions [1]. Atypical mycobacteria are known to cause other clinical syndromes such as lymphadenitis in children, lung and cutaneous infections in adults, and disseminated infections in immunosuppressed patients. They are isolated and transmitted through contaminated water. Geographically, outbreaks of atypical mycobacterial infection have been reported especially in the Rocky Mountain territories of North America [1, 2].

*Mycobacterium abscessus* (previously known as *M. chelonae*, subspecies *abscessus*) is a rare cause of chronic mastoiditis, as less than 10 cases have been reported so far. According to Redaelli de Zinis *et al*, there were only seven cases of otitis media caused by *Mycobacterium abscessus* from 1976 to 2001 [2]. *Mycobacterium abscessus* is the most pathogenic and chemotherapy-resistant rapid-growing mycobacteria commonly associated with penetrating trauma, surgery and the presence of foreign bodies. It is also one of the mycobacteria that are most often isolated from cystic fibrosis patients [3].

Infection caused by atypical mycobacterium in the petro-mastoid region causes significant diagnostic and prognostic confusion. There are no definite CT scan appearances to convince the clinician of the presence of residual infection. At this juncture, it is of value to follow up such cases with Gallium-67 (^67^Ga ) scintigraphy [4]. From a clinical standpoint, objective evidence of resolution of infection will help to guide the management of these patients. It will allow the discontinuation of prolonged antibiotic therapy, which may be expensive and potentially toxic.

The mechanism of ^67^Ga localization in inflamed and infected tissue is believed to occur through three main processes. ^67^Ga is carried bound to plasma transferrins to sites of inflammation and infection. It is also retained within the intracellular lactoferrin of leucocytes which will then migrate and accumulate at infection sites. Finally, ^67^Ga itself may be bound to the siderophores produced by pathogenic organisms localized at infection sites [4]. It localizes very early and in relatively high concentrations in the event of inflammation of the bony tissue. This makes it the preferred technique for assessing resolution of infection and to diagnose subclinical mastoiditis [4, 5]. The improvement or return to normal of ^67^Ga uptake at infection sites correlates well with the clinical inactivity of the infection.

^67^Ga-citrate also accumulates in sites of active bone remodeling but the intensity of uptake is relatively higher in the presence of infection and it is less likely to persist following resolution of infection. Thus used in combination, technetium-based agents and ^67^Ga would be useful in both diagnosing as well as in the follow-up of these cases [6].

In a study done by Strashun AM and colleagues in 1984, it was found that clinically confirmed cases of otitis media were often radiographically negative early in the course of the disease. However, radionuclide scintigraphy with technetium and gallium radiopharmaceuticals, have been demonstrated to be a more sensitive imaging modality [4]. Although CT is excellent for the localization and follow up of progression of soft tissue in the petromastoid region, it cannot be used accurately in the follow up of regression or inactivity of infection in this region [5]. This is due to the slow process of re-ossification in the skull bone.

CT is not significantly more sensitive (33%) than routine radiographic techniques. Presumably this failure of early detection is due to the clinical situation in which the inflammatory response, both soft tissue and bone, causes severe and persistent ear pain that forces the patient to seek attention only at the point in the disease where 30-50% of trabecular bone has been destroyed.

Additionally, the regional anatomy does not provide the same advantages of earlier soft-tissue changes including swelling and displacement of contiguous fat lines, swelling and obliteration of lucent planes between muscles and subcutaneous edema, relied upon radiographically to suggest the possibility of early long-bone osteomyelitis [4].

In this patient, the *Mycobacterium abscessus* isolated was resistant to co-trimoxazole. It is important to note that the patient was on a single therapy regimen for two weeks on this particular antibiotic. This was because the initial working diagnosis was chronic suppurative otitis media and co-trimoxazole is one of the antibiotics utilized in this condition. Furthermore, the culture and sensitivity of the ear swabs were not known at the initiation of therapy. Nevertheless, the initial discharge had cleared with clinical improvement after two months of treatment. It is likely that the *Mycobacterium abscessus* pathogen was initially sensitive to co-trimoxazole, however, as it was only a mono-therapy, the pathogen gradually developed resistance and the patient became symptomatic again [7].

Atypical rapidly-growing mycobacteria are resistant to conventional antimycobacterial therapy, except aminoglycosides. New drugs such as clarithromycin and erythromycin derivatives, with slightly greater activity than the parent compound and fewer side effects than erythromycin, have proven to be effective in treating *M. chelonei* infections [8]. The role of life-long antibiotic therapy is still under trial and is used at a few centres worldwide.
